# Combining Photodynamic Therapy with Immunostimulatory Nanoparticles Elicits Effective Anti-Tumor Immune Responses in Preclinical Murine Models

**DOI:** 10.3390/pharmaceutics13091470

**Published:** 2021-09-14

**Authors:** Ruben Victor Huis in ‘t Veld, Candido G. Da Silva, Martine J. Jager, Luis J. Cruz, Ferry Ossendorp

**Affiliations:** 1 Department of Radiology, Leiden University Medical Centre (LUMC), Room C2-187h, Albinusdreef 2, 2333 ZA Leiden, The Netherlands; R.V.Huis_in_t_Veld@lumc.nl (R.V.H.i.V.); C.da_Silva@lumc.nl (C.G.D.S.); 2 Department of Ophthalmology, Leiden University Medical Centre (LUMC), Albinusdreef 2, 2333 ZA Leiden, The Netherlands; m.j.jager@lumc.nl; 3 Department of Immunology, Leiden University Medical Centre (LUMC), Albinusdreef 2, 2333 ZA Leiden, The Netherlands

**Keywords:** photodynamic therapy, immunotherapy, nanoparticles, in situ vaccination, neoepitopes

## Abstract

Photodynamic therapy (PDT) has shown encouraging but limited clinical efficacy when used as a standalone treatment against solid tumors. Conversely, a limitation for immunotherapeutic efficacy is related to the immunosuppressive state observed in large, advanced tumors. In the present study, we employ a strategy, in which we use a combination of PDT and immunostimulatory nanoparticles (NPs), consisting of poly(lactic-co-glycolic) acid (PLGA)-polyethylene glycol (PEG) particles, loaded with the Toll-like receptor 3 (TLR3) agonist poly(I:C), the TLR7/8 agonist R848, the lymphocyte-attracting chemokine, and macrophage inflammatory protein 3α (MIP3α). The combination provoked strong anti-tumor responses, including an abscopal effects, in three clinically relevant murine models of cancer: MC38 (colorectal), CT26 (colorectal), and TC-1 (human papillomavirus 16-induced). We show that the local and distal anti-tumor effects depended on the presence of CD8^+^ T cells. The combination elicited tumor-specific oncoviral- or neoepitope-directed CD8^+^ T cells immune responses against the respective tumors, providing evidence that PDT can be used as an in situ vaccination strategy against cancer (neo)epitopes. Finally, we show that the treatment alters the tumor microenvironment in tumor-bearing mice, from cold (immunosuppressed) to hot (pro-inflammatory), based on greater neutrophil infiltration and higher levels of inflammatory myeloid and CD8^+^ T cells, compared to untreated mice. Together, our results provide a rationale for combining PDT with immunostimulatory NPs for the treatment of solid tumors.

## 1. Introduction

Cancer treatment currently consists of various modalities and combinations thereof, including surgical resection, radiotherapy, chemotherapy, photodynamic therapy (PDT), and immunotherapy. Interestingly, PDT can potentially serve three purposes: firstly, it can kill cancer cells directly; secondly, it can induce damage to the tumor vasculature, depending on the photosensitizer and protocols used, which lead to an impaired vascular structure or complete vascular shutdown; and thirdly, it can trigger anti-cancer immune responses [1,2]. Specifically, PDT functions by generating reactive oxygen species that subsequently damage cells in the tumor, its microenvironment, and/or its vasculature. This type of photo-ablative damage to the tumor area can induce immunogenic cell death [3,4], initiating an immune response through the exposure and/or release of damage-associated molecular patterns (DAMPs) and, in some cases, cancer (neo)antigens [3]. These DAMPs then activate diverse pattern-recognition receptors (PRRs), such as the receptor for advanced glycation end-products (RAGE), the Toll-like receptors (TLRs) TLR3/7/8/9, or absent in melanoma 2 (AIM2), among others, in dendritic cells (DCs), macrophages, epithelial, and other cells. Several DAMPs have been shown to be highly important in the immune response following PDT, including high mobility group box 1 (HMBG1) [5], surface-exposed calreticulin (CRT) [4,6,7], the surface-exposed heat shock proteins HSP70 and HSP90 [8,9,10,11,12,13], and extracellular ATP [14,15,16]. Moreover, the curative effects of PDT strongly depend on the presence of a functional adaptive immune system [15]. In this regard, we previously reported that the depletion of CD8^+^ T cells before treatment abrogates the survival benefits of PDT [17].

Cancer immunotherapy using immunostimulatory agents administered intratumorally, systemically or otherwise, has been investigated extensively. When administered intratumorally, such agents generally function by converting the tumor microenvironment and the tumor-draining lymph nodes (dLN) from an immunosuppressed *(cold)* to a pro-inflammatory *(hot)* state [18]. We previously reported that the intratumoral administration of the TLR3 ligand poly(I:C), the TLR7/8 ligand R848 and the chemokine MIP3α, co-encapsulated in a nanoparticle, is more effective in murine cancer models than either agent encapsulated alone [19,20]. Such TLR agonists are among the most potent of immunotherapies available and, accordingly, many of these agents are currently in clinical development [21]. Similarly, polyinosinic:polycytidylic acid (poly(I:C), an analog of double-stranded RNA (dsRNA) and a potent agonist of the dsRNA-sensor TLR3 [22], has been reported to inhibit the growth of certain tumors, by converting them from immunologically cold to hot [23,24,25], and to indirectly facilitate adaptive anti-tumor immune responses, through the induction of the innate immune system [26]. Additionally, poly(I:C) may also directly affect tumor cells by initiating cell death pathways, via activation of caspase-8 [27,28,29,30]. Analogously, R848, an imidazoquinolinone derivative and agonist of the single-stranded-RNA (ssRNA)-sensor TLR7/8, induces immune responses, based on signaling through MyD88 and NF-кB [31]. It induces anti-tumor responses [32], decreases the number of myeloid-derived suppressor cells (MDSCs) in tumors, and promotes the conversion of MDSCs towards a more-mature antigen-presenting phenotype [33]. Moreover, R848 has been reported to promote the polarization of tumor-associated macrophages to an M1-like phenotype, contributing to inhibition of tumor growth [34]. Similarly, TLR8 signaling has been shown to reverse suppression and inhibit the generation of senescent tumor-specific T cells and of naïve T cells [35]. Interestingly, R848 was shown to increase the expression of HMGB1, indicating a synergistic potential for combination with PDT [32]. Lastly, the chemokine MIP3α (CCL20) is a strong chemoattractant for lymphocytes [36] and acts by binding to the chemokine receptor CCR6 [37,38].

To reduce the risk of adverse systemic immune events in patients, we aim to minimize the diffusion of immunostimulatory agents from the tumor area. In this context, biocompatible nanoparticles (NPs) that can release drugs in a slow and sustained fashion are ideal vehicles for the intratumoral delivery of such agents [39], offering clear advantages over free (nude) drugs [40]. Specifically, poly(lactic-co-glycolic) acid (PLGA) NPs and liposomes are both FDA-approved vehicles and already used in the clinic [41,42]. Recently, we have reported that, in mice, PLGA-based NPs can accumulate strongly in PDT-treated tumors, compared to untreated tumors after systemic administration, providing a rationale for the combination of PDT and NP-based anti-tumor therapy [43]. Here, we report a study, in which PDT, combined with the intratumoral administration of PLGA NPs (loaded with poly(I:C), R848, and MIP3α), was analyzed for its therapeutic efficacy, compared to modality alone, in three murine cancer models: MC38 (colon adenocarcinoma model), CT26 (colon cancer carcinoma), and TC-1 (lung epithelial tumor expressing human papillomavirus (HPV)16 E6 and E7 oncoproteins). Different from studies that combine PDT with immune checkpoint inhibition [44,45,46] or indoleamine 2,3-dioxygenase inhibition [47], this approach aims to induce a proinflammatory tumor microenvironment using PDT, in addition to TLR-ligands poly(I:C) and R848, as well as the lymphocyte chemoattractant MIP3α. The antitumor efficacy of each individual immunostimulatory agent, encapsulated in a similar PLGA-NP, has been previously investigated [20]. Of relevance, each of the murine tumor models that were investigated represents a human tumor that could, potentially, be treated intratumorally in patients, via fiber optics [48,49]. In all three models, the combination treatment was highly efficacious. We observed strong anti-cancer immune responses with tumor (neo)antigen specific CD8^+^ T cells and an abscopal effect to a secondary tumor in the opposite flank. Finally, we found that our treatment modulated the immunosuppressive microenvironment into a more proinflammatory state. Together, our results indicate that a combination of PDT and intratumorally-administered PLGA NPs, loaded with immunostimulatory agents, elicits strong local and systemic anti-tumor immune responses in clinically-relevant murine models of solid tumors.

## 2. Materials and Methods

### 2.1. Materials and Reagents

PLGA (Resomer RG 502 H, lactide:glycolide molar ratio 48:52 to 52:48) was purchased from Boehringer Ingelheim, Germany. Solvents used for PLGA preparation were obtained from Sigma-Aldrich (Zwijndrecht, The Netherlands). The lipids were purchased from Avanti Polar Lipids (Alabaster, AL, USA) and included 1,2-distearoyl-sn-glycero-3-phosphoethanolamine-N-[amine (polyethylene glycol)-2000] (ammonium salt) and 1,2-distearoyl-sn-glycero-3-phosphoethanolamine-N-[methoxy(polyethylene glycol)-2000] (ammonium salt) (mPEG 2000 PE). Poly (inosinic:cytidylic acid (poly(I:C)) and the near-infrared (NIR) dye IR-780 were purchased from Sigma-Aldrich (Zwijndrecht, The Netherlands), R848 was obtained from Alexis Biochemicals (Paris, France), and MIP3α (CCL20) was purchased from R&D Systems (Minneapolis, MN, USA).

### 2.2. Preparation of PLGA-NPs

Poly-lactic-co-glycolic-acid-based NPs that encapsulate poly(I:C), R848, and MIP3α were prepared using an oil/water emulsion and the solvent evaporation-extraction method [50,51,52,53]. In brief, 200 mg of PLGA was dissolved in 3 mL of dichloromethane (DCM), containing 8 mg of poly(I:C), 4 mg of R848, and 250 μg of MIP3α, in addition to 1 mg of the NIR dye IR-780, when used for microscopy, and added drop-wise to 40 mL of aqueous 2.5% (*w*/*v*) PVA in distilled water before emulsification for 120 s, using a sonicator (250W Sonifier 250; Branson, MI, USA). After the DCM had been removed through air-drying, the lipid mPEG 2000 PE (20 mg) was dissolved in DCM and used to form a film layer on the bottom of a beaker. Subsequently, the emulsion was rapidly added to the beaker containing the lipids and the solution was homogenized for 30 s by sonification. Following overnight evaporation of the solvent at 4 °C, the PLGA NPs were collected by centrifugation at 25,000× *g* for 10 min, washed four times with distilled water, and lyophilized. The concentration of the agents entrapped by the NPs was determined by reverse phase, high-performance liquid chromatography and regression analysis, as described previously [20,54].

### 2.3. Size Distribution and Surface Charge of the NPs

The average size and zeta-potential of PLGA NPs was determined using a Zetasizer Nano ZSP (Malvern Panalytical, Malvern, UK). In brief, 50 µg of NP was dissolved in 1 mL MilliQ H_2_O, after which the size was determined by dynamic light scattering and the surface charge was measured by laser Doppler electrophoresis.

### 2.4. Cell Lines

The tumor cell line murine Colon 38 (MC38) cells on C57BL/6 background and the murine colon carcinoma cell line CT26 on BALB/c background were kindly provided by Mario Colombo and used for experiments without modification. The murine tumor cell line TC-1, expressing HPV16 E6 and E7 oncoproteins and the activated human c-Ha-ras oncogene, generated by retroviral transduction of lung fibroblasts, obtained from C57BL/6 mice, was a gift from T.C. Wu (John Hopkins University, Baltimore, MD, USA) [55]. The D1 dendritic cell (D1DCs) line, an immature splenic dendritic cell (DC) that resembles bone marrow-derived DCs [56], was cultured, as described previously [57]. All cells used were tested for mycoplasma and MAP before the onset of experiments. All tumor cell lines were cultured in culture medium, consisting of Iscove’s modified Dulbecco’s medium (IMDM; Lonza, Basel, Switzerland), supplemented with 8% fetal calf serum (Greiner, Kremsmünster, Austria), 2 mM glutamine (Gibco, Landsmeer, The Netherlands), 100 IU/mL penicillin/streptomycin (Gibco, Landsmeer, The Netherlands), and 25 μM 2-mercaptoethanol (Sigma-Aldrich, Zwijndrecht, The Netherlands) and kept in an incubator (Panasonic, ’s-Hertogenbosch, The Netherlands) at 37 °C and 5% CO_2_. For TC-1, the culture medium was further supplemented with 400 µg/mL of the selection antibiotic geneticin (G418; Thermo Fisher Scientific, Waltham, MA, USA).

### 2.5. Animal Models

All animal experiments were performed in accordance with the Code of Practice of the Dutch Animal Ethical Commission (animal permit: AVD1160020198405, approved 19 November 2019). Female BALB/c mice (6 to 12 weeks old) were purchased from Charles River (Ecully, France), and C57BL/6J mice were purchased from ENVIGO (Horst, The Netherlands). The animals were housed in the animal facility of the Leiden University Medical Center (Leiden, The Netherlands), under the specified pathogen-free conditions.

### 2.6. Photosensitizer Uptake and Retention Experiments

Photosensitizer uptake and retention were evaluated by seeding 4 × 10^4^ MC38, 3 × 10^4^ CT26, or 2.5 × 10^4^ TC-1 cells in separate wells of a 24-well plate (Corning, Glendale, CA, USA) in culture medium and subsequent incubation overnight at 37 °C and 5% CO_2_. For the uptake experiments, cells were incubated with indicated concentrations of Radachlorin^®^ (Radapharma International, Loon op Zand, The Netherlands) for a specified time. Following incubation, the cells were washed 3 times with PBS and fixed in phosphate buffered saline (PBS) containing 1% formalin (J.T. Baker, Landsmeer, The Netherlands) at 4 °C for 15 min. The fixative was then washed away with PBS, after which the cells were reconstituted in a fluorescence-activated cell sorting (FACS) buffer (PBS with 0.5% bovine serum albumin (BSA) and 0.02% sodium azide). The fluorescence of the photosensitizer was used to determine its uptake, using flow cytometry on an LSR II (BD Biosciences, San Jose, CA, USA). For the retention experiment, cells incubated with photosensitizer for 4 h were washed 3 times in PBS and supplied with fresh culture medium. After an indicated amount of time, the samples were washed 3 times in PBS, fixed in 1% formalin (J.T. Baker, Landsmeer, The Netherlands) at 4 °C for 15 min before washing in PBS, reconstituting in FACS buffer, and analyzed by flow cytometry.

### 2.7. PDT In Vitro Cytotoxicity

For PDT in vitro, 4 × 10^4^ MC38, 3 × 10^4^ CT26, and 2.5 × 10^4^ TC-1 cells were seeded in 24-well plates (Corning, Glendale, CA, USA) in culture medium and kept overnight at 37 °C and 5% CO_2_. Cells were then incubated with 2 µM Radachlorin^®^, unless indicated otherwise, for a specified amount of time, washed 3 times with PBS and supplied with 500 µL fresh medium. Illumination was performed at a light intensity (fluence rate) of 116 mW/cm^2^, for a total light dose (fluence) of 20 J/cm^2^, using a 662 nm Milon Lakhta laser, unless indicated otherwise. The following day, the cells were collected in FACS buffer, stained with annexin V-FITC (BD Biosciences, San Jose, CA, USA) at 3 µL per sample and 0.5 µM 4′,6-diamidino-2-phenylindole (DAPI) (Sigma-Aldrich, Zwijndrecht, The Netherlands) in annexin V-binding buffer (0.1 M 4-(2-hydroxyethyl)-1-piperazineethanesulfonic acid (HEPES), 1.4 M NaCl, and 25 mM CaCl_2_ in deionized water with a pH set to 7.4. sterile filtered using a 0.2 µm filter) and, finally, analyzed by flow cytometry. As a positive control, cells were subjected to three freeze/thaw cycles at −20 °C before staining and analysis by flow cytometry.

### 2.8. Maturation of D1DCs after Incubation with NPs

The biological activity of the NP-encapsulated agents was evaluated by seeding 5 × 10^4^ D1DCs in 96-well plates (Corning, Glendale, CA, USA) and incubated with the NPs for 48 h in an incubator. The NP concentrations were matched to poly(I:C) at 5 µg/mL and serially diluted, according to annotated concentrations, to establish a dose-response curve and enable comparison with the free ligand at 5 µg/mL. The cells were stained for the DC maturation markers CD86 and CD40, using anti-CD86-APC (clone GL1; eBioscience, Waltham, MA, USA) and anti-CD40-PE (clone 1C10; eBioscience, Waltham, MA, USA), respectively, and expression was measured by flow cytometry. The supernatant was collected, after which IL12 was analyzed by a standard sandwich ELISA, using the purified anti-mouse IL12/IL23 p40 (clone C15.6; Biolegend, San Diego, CA, USA) and biotin-labelled anti-mouse IL12/IL23p40 antibodies (clone C17.8; Biolegend, San Diego, CA, USA). The plates were read at 450 nm, using a Bio-Rad 680 microplate reader (Bio-Rad Laboratories, Veenendaal, The Netherlands).

### 2.9. Toxicity of the NPs 

The toxicity of the NPs to MC38, CT26, and TC-1 cells was determined by seeding 5 × 10^4^ cells in 96-well plates (Corning, Glendale, CA, USA) and incubating them with the NPs in a range of concentrations (6.25 µg/mL to 200 µg/mL) for 72 h. Cell viability was measured by adding 3-(4,5-dimethylthiazol-2-yl)-5-(3-carboxymethoxyphenyl)-2-(4-sulfophenyl)-2H-tetrazolium (MTS) reagent, according to manufacturer instructions (Abcam, Cambridge, UK), and absorption was measured at 490 nm on a Bio-Rad iMark microplate absorbance reader (Bio-Rad Laboratories, Veenendaal, The Netherlands) after incubation.

### 2.10. Maturation of D1DCs after Incubation with PDT-Treated Tumor Cells

The immunostimulatory effects of PDT were preliminarily ascertained in a cellular assay involving dying PDT-treated cells and D1 dendritic cells. Firstly, 10^4^ D1DCs were seeded in 96-well plates (Corning, Glendale, CA, USA) and incubated for 24 h. The following day, tumor cells were incubated with 2 µM Radachlorin^®^ for 4 h (as described in 2.7) and treated with PDT at 116 mW/cm^2^ for 20 J/cm^2^. These (dying) treated tumor cells were then added to the D1DCs at a ratio of 20:1 (tumor cell/D1DC), after which the cells were co-incubated for 24 h in an incubator. The cells were then collected and stained with 0.5 µM DAPI (Sigma-Aldrich, Zwijndrecht, The Netherlands), CD11c-APC-Cy7 (clone N418; Thermo Fisher Scientific, Waltham, MA, USA), MHC-II-PE (H-2kb AF6-88.5; BD Biosciences, San Jose, CA, USA), CD86-FITC (clone GL1; eBioscience, Waltham, MA, USA), and analyzed by flow cytometry on an LSR-II (BD Biosciences, San Jose, CA, USA). Live D1DCs were gated, based on DAPI^-^CD11c^hi^ after size/morphology and doublet exclusion, based on FSC/SCC patterns.

### 2.11. PDT and NP Tumor Treatments In Vivo

For PDT in vivo, C57BL/6J mice were inoculated with 0.5 × 10^6^ MC38 or 1 × 10^5^ TC-1 cells in 200 µL PBS and BALB/c mice were inoculated with 0.2 × 10^6^ CT26 cells in 200 µL PBS, on the left and/or right flanks, as indicated per experiment. Once the tumors had reached an average volume of approximately 125 mm^3^, the mice were randomly divided into groups and treated with PDT, as described previously [17,58]. Briefly, 20 mg/kg Radachlorin^®^ was administered intravenously into the tail vein and allowed to distribute for 6 h. Then, the skin surrounding the tumor area was shaved before illumination under isoflurane anesthesia at a fluence rate of 116 mW/cm^2^ over 1000 s for a fluence of 116 J/cm^2^. The next day, the mice were injected intratumorally with NPs at concentrations corresponding to 2.5 mg/kg (50 μg) poly(I:C), at 0.7 mg/kg (14 μg) of R848, and 0.05 mg/kg (1 μg) of MIP3α in a total volume of 30 µL per treatment. These intratumoral injections were repeated every other day for a total of four treatments for the MC38 and CT26 models, as well as a total of two treatments for the TC-1 model. From this point onwards, the tumor growth was measured regularly until the end of the experiment.

### 2.12. Detection of Blood Tetramers

The capacity of PDT and the NPs to induce antigen-specific T cells in the blood of TC-1 tumor-bearing mice was determined by analyzing (25 µL) blood obtained from the tail vein at day 8 after PDT. Red blood cells were removed using a lysis buffer, after which the cells were incubated with an APC-labeled, MHC class I (H-2Db) HPV16 E749-57 (RAHYNIVTF) (H-2Db) tetramer. Next, the cells were stained with anti-CD8α-PE (clone 53-6.7; eBioscience, Waltham, MA, USA), anti-CD3-eFluor 450 (clone 17A2; eBioscience, Waltham, MA, USA), and analyzed by flow cytometry on an LSR-II (BD Biosciences, San Jose, CA, USA). Gating of CD8^+^ T cells was based on CD3^+^CD8^+^ events after size/morphology and doublet exclusion was based on FSC/SCC patterns.

### 2.13. Depletion of CD8^+^ Cells

Mice were treated with 1 mg/kg (20 µg) anti-CD8-depleting antibodies via subcutaneous injection (clone 2.43; Leiden University Medical Center, Leiden, The Netherlands) in 100 µL PBS every 7 days, starting one day before treatment. Circulating CD8^+^ T cells were qualified by analyzing blood (50 µL) obtained from the tail vein the morning before treatment. Red blood cells were removed using lysis buffer, after which the cells were stained with anti-CD8α-PE (clone 53-6.7; eBioscience, Waltham, MA, USA), and then anti-CD3-eFluor 450 (clone 17A2; eBioscience, Waltham, MA, USA), and analyzed by flow cytometry on an LSR-II (BD Biosciences, San Jose, CA, USA).

### 2.14. Analysis of the Tumor Microenvironment, Draining Lymph Node and Spleen

Immunocompetent mice were inoculated with tumor cells in the right and left flanks, and then subcutaneously injected with CD8-depleting antibodies one day before tumors became established. When the tumors were established (~125 mm^3^), PDT was performed on one tumor by administration of 20 mg/kg Radachlorin in the tail vein and irradiating with 662 nm light at a drug-to-light interval of 6 h at 116 mW/cm^2^ for 116 J/cm^2^. The next morning, animals were intratumorally injected with the NPs at an interval of 2 days. The day following the second NP administration, the mice were sacrificed, after which the tumors, tumor-draining lymph node of the treated tumor, and the spleen were harvested, processed, and stained for analysis by flow cytometry. Tumors were excised, and then incubated with Liberase protease mix (Sigma-Aldrich, Zwijndrecht, The Netherlands) for 15 min to 30 min at 37 °C. Liberase-treated tumor fragments, spleens, and lymph nodes were processed through a cell strainer (Corning, Glendale, CA, USA) to obtain single-cell suspensions. The samples were washed 2 times with culture medium and then washed 2 times with FACS buffer. Samples were stained with antibody mixes (see below) for analysis by flow cytometry. All flow cytometric analyses were performed on samples provided in FACS buffer on a Cytek Aurora 5-laser flow cytometer (Cytek, Fremont, CA, USA). The myeloid antibody panel consisted of CD11b-eFluor450 (clone M1/70; Thermo Fisher Scientific, Waltham, MA, USA), Ly6C-BV605 (clone HK1.4; Biolegend, San Diego, CA, USA), F4/80-FITC (clone BM8; Biolegend, San Diego, CA, USA), Ly6G-AF700 (clone 1A8; Biolegend, San Diego, CA, USA), CD45.2-APC-eFluor780 (clone 104; Thermo Fisher Scientific, Waltham, MA, USA), and 7AAD (Invitrogen, Waltham, MA, USA) viability staining. The lymphoid antibody panel consisted of CD44-V450 (IM7; Thermo Fisher Scientific, Waltham, MA, USA), CD3e-FITC (Clone 145-2C11; Thermo Fisher Scientific, Waltham, MA, USA), CD4-APC (clone RM4-5; Thermo Fisher Scientific, Waltham, MA, USA), CD8α-APC-R700 (clone 53-6.7; BD Biosciences, San Jose, CA, USA), CD45.2-APCeFluor780 (clone 104; Thermo Fisher Scientific, Waltham, MA, USA), and 7AAD viability staining (Invitrogen, Waltham, MA, USA).

### 2.15. Intracellular Cytokine Staining

Single-cell suspensions of splenocytes, obtained as in 2.14, were incubated with D1DCs that were loaded overnight with 5 µM synthetic peptides of the MC38 neoepitopes Adpgk (peptide sequence: HLELASMTNMELMSSIVHQ) and Rpl18 (peptide sequence: KAGGKILTFDRLALESPK) [59,60], in presence of 2 µg/mL Brefeldin A for 8 h at 37 °C. The samples were then stained with antibody mixes for flow cytometry. Again, all cytometric analyses were performed on samples provided in FACS buffer on a Cytek Aurora 5-laser flow cytometer. The antibody panel consisted of granzyme B-V450 (clone NGZB; Thermo Fisher Scientific, Waltham, MA, USA), CD3-BV510 (clone 145-2C11; BD Biosciences, San Jose, CA, USA), TNFα-FITC (clone MP6-XT22; Thermo Fisher Scientific, Waltham, MA, USA), IL-2-PE (clone JES6-5H4; Thermo Fisher Scientific, Waltham, MA, USA), IFN-γ-PE-Cy7 (clone XMG1.2; BD Biosciences, San Jose, CA, USA), CD8α-APC-R700 (clone 53-6.7; BD Biosciences, San Jose, CA, USA), and 7AAD viability staining (Invitrogen, Waltham, MA, USA).

### 2.16. Statistics 

Graph Pad Prism software (version 8) was used for statistical analysis. Data were analyzed as indicated for individual experiments.

## 3. Results

### 3.1. PDT In Vitro

**Figure 1:**
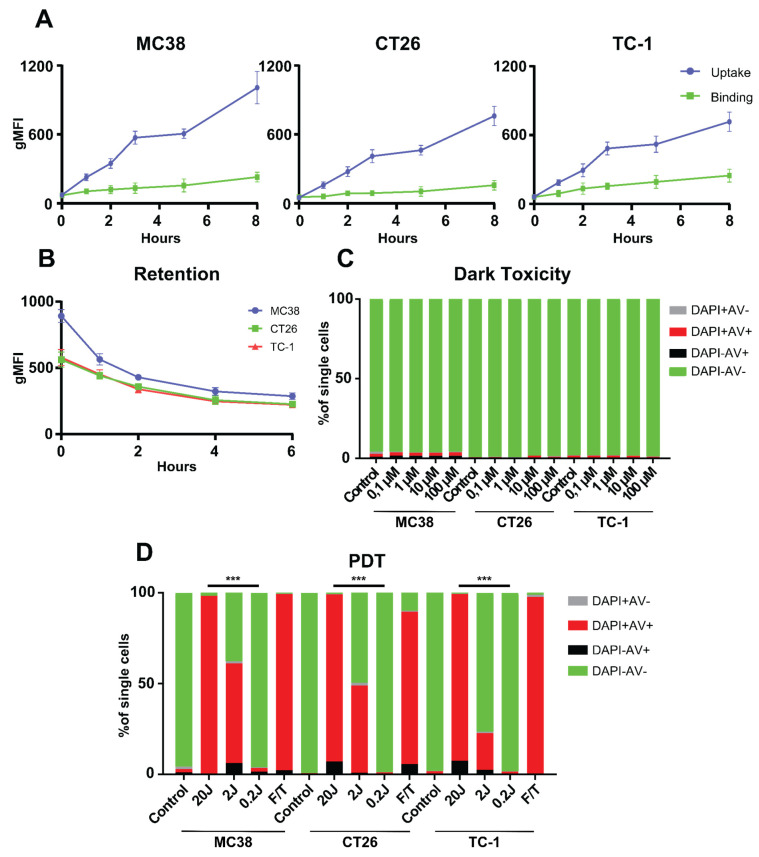
Cellular uptake, binding, retention, and cytotoxicity of the photosensitizer Radachlorin, in three clinically relevant tumor cell lines. (**A**) Cellular uptake and binding assays with the photosensitizer Radachlorin (2 µM) in MC38, CT26, and TC-1 cells over time. The uptake and binding assays were performed by incubating cells with photosensitizer at 37 °C and 4 °C, respectively. Detection was performed by flow cytometry using the fluorescence of Radachlorin and represented as geometric mean fluorescence intensity (gMFI). (**B**) Retention of Radachlorin (2 µM) in MC38, CT26, and TC-1 cells after a pulse of 4 h, washing and detection by flow cytometry, showing the gMFI. (**C**) Dark toxicity after incubation with Radachlorin (0.1 µM to 100 µM) for 4 h, followed by washing and incubation overnight. Cells were stained with DAPI and annexin V-FITC to determine cell viability by flow cytometry. (**D**) Cytotoxicity of Radachlorin (2 µM) treatment followed by PDT. Cells were incubated for 4 h, after which they were washed and irradiated with 662 nm light 116 mW/cm^2^ (0.2 J/cm^2^ to 20 J/cm^2^). Three freeze/thaw cycles at −20 °C were used as positive control. The next day, the cells were stained with DAPI and annexin V-FITC to determine their viability by flow cytometry. All data shown consist of an average of three independent experiments. Statistical analysis was performed using the Students *t*-test, by comparing experimental groups at the indicated timepoints (*** *p* < 0.0001).

We previously studied and characterized, PDT with the photosensitizer (PS) Radachlorin reported strong induction of anti-tumor responses and disruption of the tumor vasculature in the MC38 tumor model [43]. In the current study, we used flow cytometry to show that Radachlorin is internalized by MC38, CT26, and TC-1 cells over time by measuring the geometric mean fluorescence intensity (gMFI), with uptake increasing up to 8 h post-incubation (Figure 1A). Binding of the PS, as investigated by incubation at 4 °C, induces a markedly lower fluorescent signal, when compared to the uptake in all three tested cell lines over time (Figure 1A), indicating that the majority of the PS is, indeed, taken up by the cells. Furthermore, the PS was shown to stay inside the cells, up to at least 6 h post-pulse (Figure 1B). Moreover, we confirmed that Radachlorin was non-toxic to all the tested cell lines after incubation, in the absence of light (dark toxicity), at Radachlorin concentrations from 0.1 µM to 100 µM (Figure 1C). We investigated the effect of in vitro PDT on cell viability after 4 h of incubation at 2 µM Radachlorin, followed by illumination with 662 nm laser light, at a fluence rate of 116 mW/cm^2^ for a fluence of 20 J/cm^2^. Flow cytometry, based on staining for the death marker DAPI and early-apoptotic marker annexin V (Figure 1D), was subsequently applied on the treated cells. The single PDT treatment-induced near-complete cell death, comparable to three freeze/thaw cycles at −20 °C. Importantly, at 2 J/cm^2^, approximately 61 ± 4% of MC-38, 49 ± 3% of CT26, and 23 ± 6% of TC-1 cells were stained by annexin V and/or DAPI, indicating differences in sensitivity to PDT among tumor cell lines. PDT-induced cell death diminished with decreasing fluence: at a fluence of 0.2 J/cm^2^, we observed levels of cell death comparable to those in the untreated tumor cells. Together, our results indicate: that the photosensitizer Radachlorin is gradually internalized by MC38, CT26, and TC-1 tumor cells in vitro; that it remains in these cells for up to 6 h post-incubation; that it does not exhibit dark toxicity; and that, following PDT, it kills cells from all three tumor lines, at levels similar those obtained by multiple freeze/thaw cycles.

### 3.2. Radachlorin PDT Induces Immunogenic Cell Death

**Figure 2:**
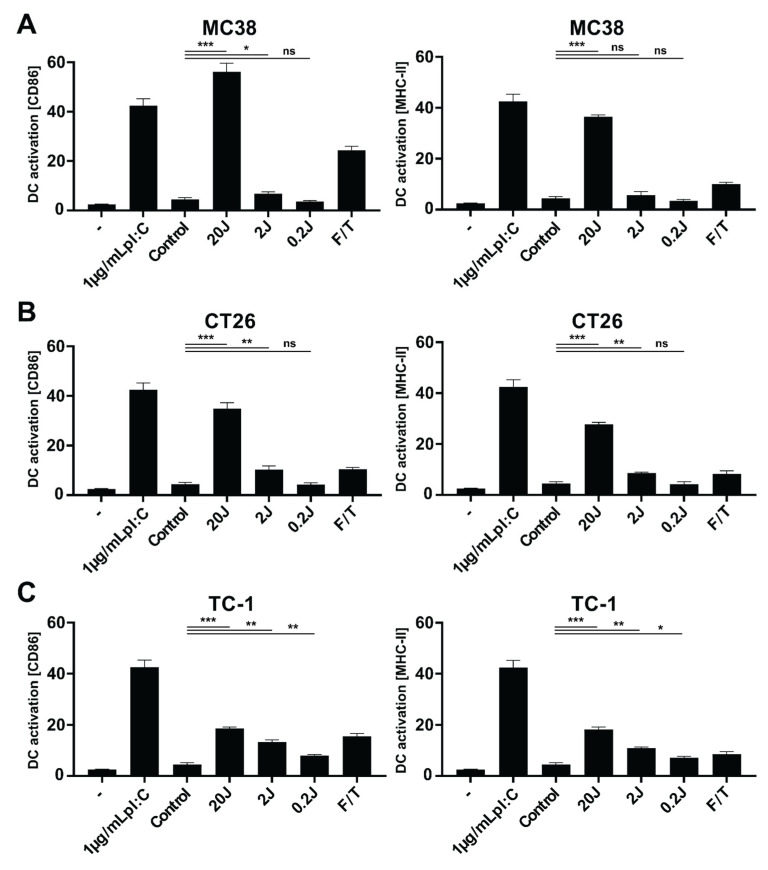
Immune stimulating effects of PDT-induced cancer cell death on dendritic cells. MC38 (**A**), CT26 (**B**), or TC-1 (**C**) cells were treated by PDT after 4 h of incubation with Radachlorin (2 µM) at 116 mW/cm^2^ (0.2 J/cm^2^ to 20 J/cm^2^) or three freeze/thaw (F/T) cycles at −20 °C, incubated with murine DCs for 24 h immediately post-treatment. The percentage of CD86^hi^ and MHC-II^hi^ cells in live DCs (CD11c^+^DAPI^−^ cells) were compared to untreated DCs (-), to DCs incubated with poly(I:C) (1 µg/mL), and to DCs incubated with untreated MC38 (control). Data from three independent assays shown as a mean ± SD. All data shown consist of an average of three independent experiments. Statistical analysis was performed using the Students *t*-test, by comparing experimental groups to control (* *p* < 0.05, ** *p* < 0.01 and *** *p* < 0.0001).

Next, we investigated the immunological effects of PDT-induced cancer cell death on the maturation of dendritic cells (DCs). To this end, DCs were incubated for 24 h with PDT-treated cancer cells and evaluated for expression of the maturation markers CD86 and MHC-II by flow cytometry. For MC38 cells, the protocol that induced the strongest cell death also induced the greatest upregulation of both markers at levels higher than those observed for the positive control, three freeze/thaw cycles (Figure 2A). Moreover, the PDT-treated cancer cells induced upregulation of the maturation markers at levels comparable to treatment with 1 µg/mL of the TLR3 ligand poly(I:C), an immunostimulatory agent that induces strong upregulation of these markers. A similar trend was observed for CT26, although the upregulation of the markers was lower than for MC38 (Figure 2B). Finally, incubation of the DCs with TC-1 cancer cells induced a slight upregulation of the maturation markers, with levels only slightly increased, compared to incubation with the positive control of three freeze/thaw cycles (Figure 2C). Taken together, these results suggest that PDT treatment of MC38 and CT26 cells, and to a much smaller extent of TC-1 cells, leads to strong upregulation of maturation markers on DCs in vitro.

### 3.3. Physicochemical Characterization and Biological Activity of PLGA-PEG (poly(I:C), R848, MIP3α) NPs

We previously characterized the PLGA-based NPs that we used in this study for the local, slow, and sustained release of poly(I:C), R848 and MIP3α for size, zeta-potential, TEM morphology, stability, drug release kinetics, uptake, cytotoxicity, DC maturation, and chemoattractant capacity [19,20]. Moreover, we reported, in another study, on the immunological effects of NP-encapsulated poly(I:C), R848, and of MIP3α, either combined or separate, in MC38 and TC-1 models [20]. For the current study, we re-analyzed an aliquot of NPs from the pooled production batches. The NPs exhibited an average size of 249.6 nm, as evaluated by dynamic light scattering (Supplementary Materials Figure S1A and Table 1) and an average zeta-potential (ζ) potential of −21.4mV, as determined using a Zetasizer (Supplementary material Figure S1B and Table 1).

To determine whether the biological activity of the encapsulated compounds had been preserved during NP synthesis and storage, we incubated the NPs with DCs in a range of concentrations (0 µg/mL to 5 µg/mL poly(I:C)). The expression of the maturation markers, CD40 and CD86, was evaluated after incubation with the NPs: both maturation markers were upregulated at levels comparable to that observed for treatment with free (nude) poly(I:C) added at equimolar concentration (Supplementary material Figure S1C), indicating that the encapsulated compounds had retained their biological activity. Further corroborating the immunostimulatory activity of their cargo, the NPs also induced production of IL-12 at similar levels to that observed for the treatment with free poly(I:C) (Supplementary material Figure S1D). The toxicity of the NPs was evaluated by MTS assay, after incubation at concentrations of 0 µg/mL to 200 µg/mL. The NPs did not exhibit any direct cytotoxicity to MC38 (Supplementary material Figure S1E), CT26 (S1F), or TC-1 (Supplementary material Figure S1G) cells, even at the highest concentration tested. Together, these results suggest that the NPs have favorable size and charge distributions, retain the immunostimulatory activity of their cargo, and are non-toxic to tumor cells.

The combination of PDT and immunostimulatory NPs strongly inhibits tumor growth and induces anti-tumor immune responses in vivo.

**Figure 3:**
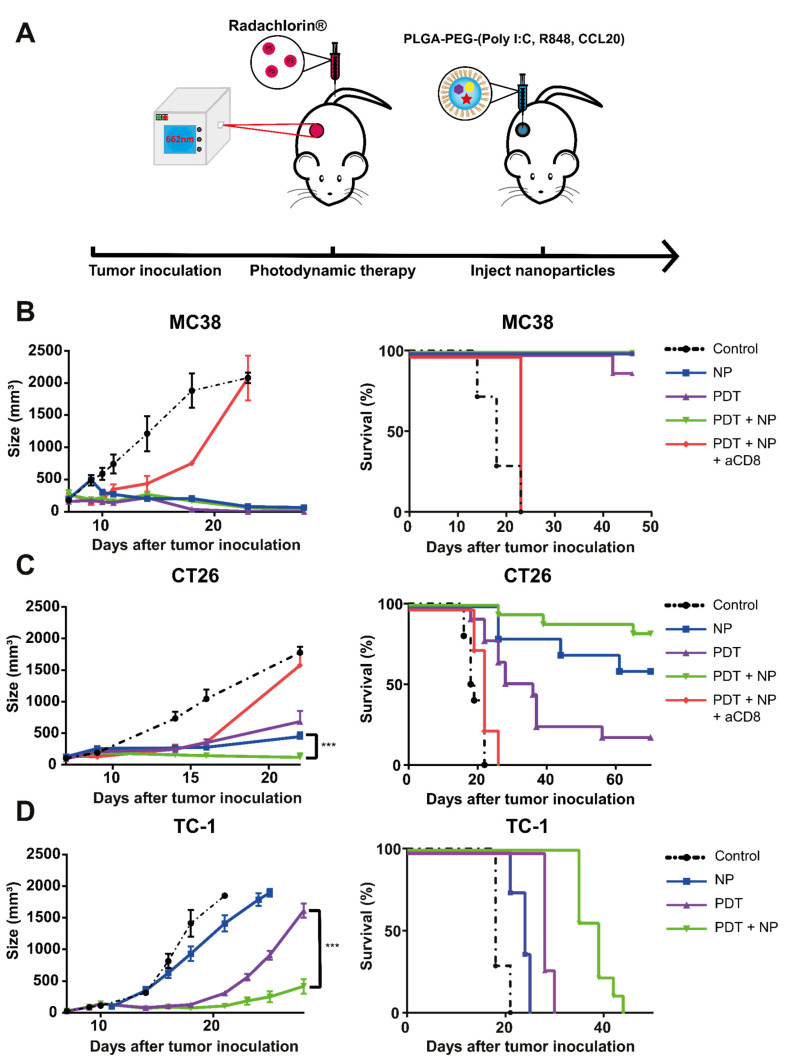
Anti-tumor efficacy of PDT combined with immunostimulatory NPs in mice bearing MC38, CT26, or TC-1 tumors. (**A**) Description of the protocol: immunocompetent mice were inoculated with tumor cells in the right flank (*n* ≥ 10 mice per group). CD8-depleting antibodies were injected 1 day before treatment. Once the tumors had become established (~125 mm^3^), the mice were treated with PDT, by administering Radachlorin (20 mg/kg) via a tail-vein injection, followed by irradiation (662 nm) at a drug/light interval of 6 h, at 116 mW/cm^2^ for 116 J/cm^2^. The next morning, treatment with NPs was started, with an interval of 2 days for a total of four (MC38 and CT26) or two (TC-1) i.t. administrations. (**B**) Tumor-growth and survival curves for C57BL/6J mice bearing MC38 tumors. (**C**) Tumor-growth and survival curves for BALB/c mice bearing CT26 tumors. (**D**) Tumor-growth and survival curves for C57BL/6J mice bearing TC-1 tumors. Statistical analysis was performed using the Students *t*-test, by comparing experimental groups at the indicated timepoints (*** *p* < 0.0001).

Next, we assessed the tumor-debulking capacity of PDT and the immunostimulatory effects of the NPs, separately and combined, in mice bearing MC38, CT26, or TC-1 tumors. Thus, mice with established tumors (average volume: ~125 mm^3^) were treated with PDT after a drug-to-light interval of 6 h, with 662 nm light at a fluence rate of 116 mW/cm^2^, for a fluence of 116 J/cm^2^ (Figure 3A). A DLI of 6h was chosen to ensure an efficient disruption of the tumor and its surrounding vasculature, as shown previously by intravital microscopy. The debulking effects of this PDT treatment on the tumor mass were pronounced in all three models, although the duration of the delay in tumor growth varied. The PDT treatment eradicated all MC38 tumors (Figure 3B), approximately half of the CT26 tumors resumed growth at a slow rate after 10 days (Figure 3C), while all the TC-1 tumors resumed growth after 10 days (Figure 3D). Treatment with intratumoral injections of NPs, with the three immunostimulatory agents, induced strong anti-tumor responses in the MC38 and CT26 models (Figure 3B,C); however, it showed little effect on the TC-1 model (Figure 3D). The combination of PDT and the NPs was as effective as PDT alone and as NPs alone in the MC38 model, as both treatments alone induced near-complete cures (Figure 3B); however, the combination showed superior efficacy to either treatment alone in the CT26 model, as the tumors remained in regression 10 days after co-treatment and induced an enhanced survival rate up to 70 days post-treatment (Figure 3C). Furthermore, the combination treatment significantly delayed tumor growth in the TC-1 model, initially similar to PDT alone; however, the TC-1 tumor growth developed at a much slower rate three weeks after the co-treatment than those treated with PDT alone did (Figure 3D). The weight of the animals was not significantly affected by the treatment in all models, indicating an acceptable level of treatment-induced toxicity (Supplementary Materials Figure S2A–C). In cancer immunotherapy, CD8^+^ T cells are often central in successful tumor clearance. Therefore, we investigated the importance of this population in our setting by administering CD8-depleting antibodies, starting one day before PDT treatment and subsequently administering them periodically for the remainder of the experiment. For mice bearing MC38 or CT26 tumors, pre-treatment depletion of their CD8^+^ cells (Supplementary Materials Figure S3A,B) led to rapid tumor growth, after an initial delay in growth that had directly followed treatment. Together, the above results demonstrate that the combination of PDT and immunostimulatory NPs in tumor-bearing mice induces strong, CD8-dependent anti-tumor immune responses, with near-complete survival of MC38, strongly enhanced survival of CT26, and a delay in growth of TC-1 tumors.

### 3.4. The Combination of PDT and NP Elicits a CD8^+^ T cell-Dependent Abscopal Effect in Mice Bearing Bilateral MC38 or CT26 Tumors

**Figure 4:**
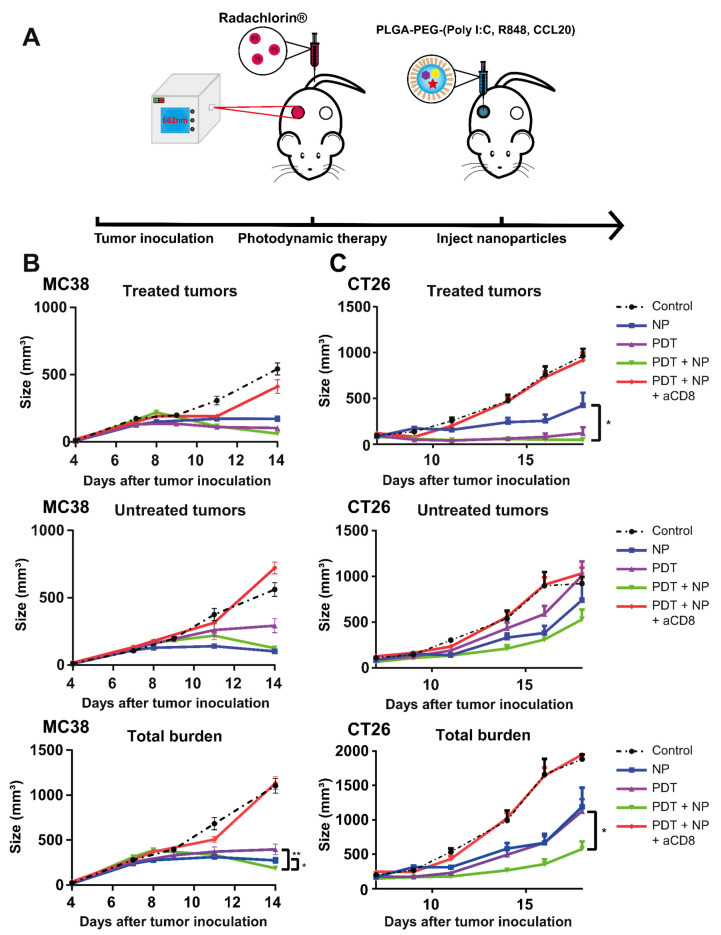
The combination of PDT and NP induces an abscopal effect in mice bearing bilateral MC38 or CT26 tumors. (**A**) Description of the protocol: immunocompetent mice were inoculated with tumor cells in the right and left flanks (*n* ≥ 9 mice per group) and injected with CD8-depleting antibodies 1 day before treatment. Once the tumors had become established (~125 mm^3^), the mice were treated with PDT by administering Radachlorin (20 mg/kg) via a tail-vein injection, followed by irradiation (662 nm) at a drug/light interval of 6 h, at 116 mW/cm^2^ for 116 J/cm^2^. The next morning, the mice were treated with NPs at an interval of 2 days for a total of four administrations. (**B**) Tumor-growth curves of the treated tumors (upper panel), untreated tumors (middle panel), and total tumor burden (lower panel) for C57BL/6J mice bearing MC38 tumors. (**C**) Tumor-growth curves of the treated tumors (upper panel), untreated tumors (middle panel), and total tumor burden (lower panel) tumors for BALB/c mice bearing CT26 tumors. Statistical analysis was performed using the Students *t*-test, by comparing experimental groups at the indicated timepoints (* *p* < 0.05, ** *p* < 0.01).

To study the induction of an abscopal effect by the treatments in the MC38 and CT26 models, we inoculated mice with two tumors, one on each opposing flank, and then treated only one of the tumors, using the same protocol as above, for mice bearing a single tumor (Figure 4A). For MC38, both the separate and combination treatment induced a delay in tumor growth on the untreated tumors, compared to the control (untreated) mice, with the NP treatment and the combination treatment showing the strongest effects (Figure 4B). At 14 days post-inoculation, the combination induced an enhanced tumor growth inhibition on the total tumor burden, compared to either treatment alone (Figure 4B), consequently extending the survival, compared to PDT alone or control (untreated) but not versus NP alone (Supplementary Materials Figure S4A). A similar tumor growth delay was observed for the CT26 model (Figure 4C). As in MC38, the NP treatment and the combination treatment induced the greatest effect in CT26, whereby PDT and the combination treatment induced the strongest effects on the treated tumors (Figure 4C). The combination treatment induced an enhanced tumor growth inhibition on the total tumor burden, when compared to PDT or NP alone (Figure 4C); however, as in MC38, it only provided superior survival relative to the control (untreated) (Supplementary material Figure S4B). As in the unilateral models, the weight of the animals was not significantly affected by the treatment in both tumor models, indicating an acceptable level of treatment-induced toxicity (Supplementary Materials Figure S5A,B). Importantly (and consistent with our previous results from the single-tumor experiments), CD8^+^ T cells were essential for greater survival of the treated groups; thus, in mice bearing bilateral MC38 (Figure 4A) or CT26 (Figure 4C) tumors, the benefits of the combination treatment on survival are completely abrogated after depletion of CD8^+^ T cells. Together, these results show that the combination of PDT and immunostimulatory NPs provides superior systemic anti-tumor immune responses in mice bearing bilateral MC38 or CT26 tumors, compared to either treatment alone.

### 3.5. The Combination of PDT and Immunostimulatory NPs Provides Enhanced, Tumor-Specific Immune Responses in Mice Bearing Bilateral MC38 or TC-1 Tumors

**Figure 5:**
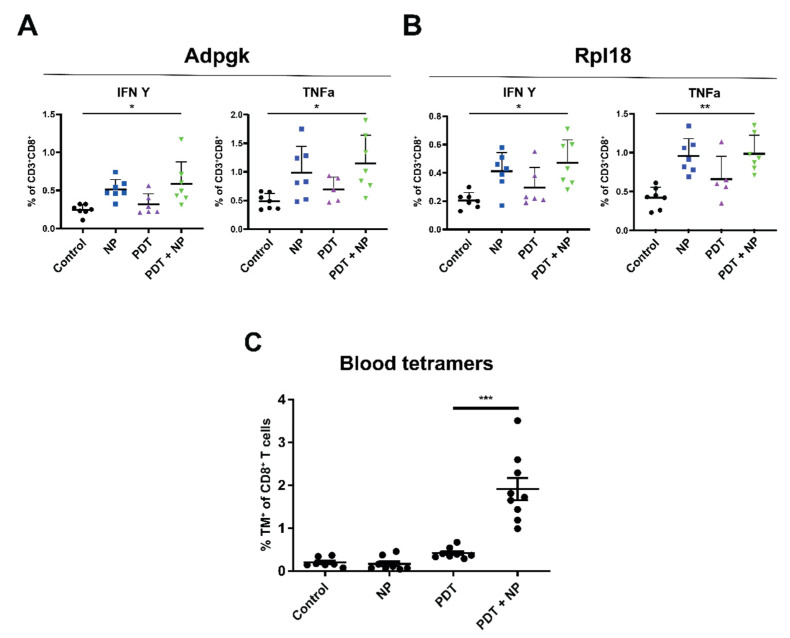
The combination of PDT and immunostimulatory NPs induces enhanced, tumor-specific immune responses. Description of the protocol: immunocompetent mice were inoculated with tumor cells in the right and left flanks (*n* ≥ 5 mice per group) and injected with CD8-depleting antibodies 1 day before treatment. Once the tumors had become established (~125 mm^3^), the mice were treated with PDT, by administering Radachlorin (20 mg/kg) via a tail-vein injection, followed by irradiation (662 nm) at a drug/light interval of 6 h, at 116 mW/cm^2^ for 116 J/cm^2^. The next morning, the mice were treated with NPs, at an interval of 2 days for a total of two administrations. The day following the second NP administration, the mice were sacrificed, and their spleens were collected and processed for further analysis. Isolated splenocytes were incubated with D1DCs, loaded with the MC38 neoepitopes Adpgk (**A**) or Rpl18 (**B**) in the presence of Brefeldin A, after which, the CD8^+^ T cells were analyzed for production of intracellular cytokines. (**C**) Evaluation of tumor-antigen-specific CD8^+^ T cells in the blood of C57BL/6J mice bearing a single TC-1 tumor at day 8 post-treatment. Tumor-antigen-specific CD8^+^ T cells were stained with APC-labeled HPV16 E749-57 (RAHYNIVTF) MHC class I (H-2Db) tetramers and detected by flow cytometry. Significance was determined using the Mann-Whitney U test (* *p* < 0.01; ** *p* < 0.001, *** *p* < 0.0001).

PDT-induced tumor cell death has been suggested to promote the exposure of previously inaccessible (neo)epitopes, which could then trigger tumor-specific immune responses. Accordingly, PDT could simultaneously function both as a direct tumor-killing modality and as an in-situ vaccination strategy. We reasoned that the immunostimulatory effects of PDT might be enhanced through combination with immunostimulatory NPs, which would serve as a potent adjuvant to facilitate tumor-specific T cell activity. To explore this hypothesis, we inoculated mice with one tumor on each flank, and then treated only one of the tumors with the combination of PDT and NP, as described above (Figure 4A). The day following the second NP administration, the mice were sacrificed, and the organs were subsequently collected and processed for further analysis. The presence of tumor-specific T cells among splenocytes, obtained from these mice, was investigated by stimulation, with D1 dendritic cells preloaded with the MC38 neoepitopes Adpgk or Rpl18 [60], and subsequent analysis of intracellular cytokine production. Interestingly, splenocytes from the mice treated with the combination exhibited greater levels of CD8^+^ T cells positive for IFN γ and TNFα after incubation with Adpgk (Figure 5A) or Rpl18 (Figure 5B). These results indicate that the combination can enhance specific anti-tumor immune responses, for which the NPs appear to have a stronger effect than PDT. Furthermore, for the TC-1 model, we measured the HPV-E7-specific CD8^+^ T cells in blood 8 days post-treatment and observed a considerably higher number of these cells in the animals that had been treated with the combination than in those treated with either single treatments or in the control (untreated) mice (Figure 5C). Together, these results suggest that the combination enhances MC38-neoepitope-specific CD8^+^ T cells in the spleen, induces high circulating levels of TC-1-specific CD8^+^ T cells, and that these effects are superior, compared to those observed for either PDT or immunostimulatory NPs alone.

### 3.6. The Combination Treatment Induces an Inflammatory State in Colon Tumors in Mice

**Figure 6:**
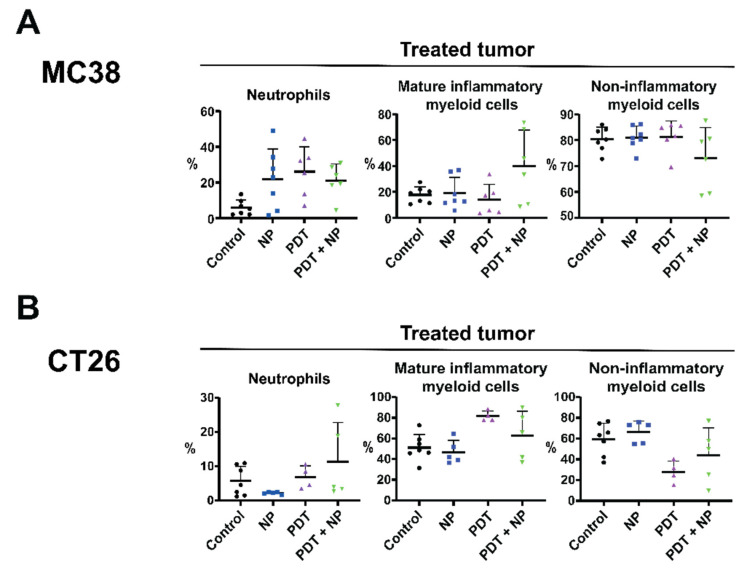
The combination of PDT and immunostimulatory NPs induces an inflammatory state in the tumor microenvironment. Immunocompetent mice were inoculated with bilateral MC38 or CT26 tumors (*n* ≥ 5 mice), in the right and left flanks (*n* ≥ 5 mice per group). Once the tumors had become established (~125 mm^3^), the mice were treated with PDT by administering Radachlorin (20 mg/kg), via a tail-vein injection, followed by irradiation (662 nm) at a drug/light interval of 6 h, at 116 mW/cm^2^ for 116 J/cm^2^. The next morning, the mice were treated with the immunostimulatory NPs, at an interval of 2 days, for a total of two administrations. The day following the second NP administration, the mice were sacrificed, after which the tumors were collected, processed, and stained for analysis by flow cytometry. Cell populations are shown in percentages for mice bearing bilateral MC38 (**A**) or CT26 (**B**) tumors. Gating was performed in FlowJo and included only living (7AAD^−^) CD45.2^+^ cells. Populations were further gated to include neutrophils (CD11b^+^Ly6G^+^), mature inflammatory myeloid cells (CD11b^+^CD86^+^Ly6C^hi^), and non-inflammatory myeloid cells (CD11b^+^Ly6C^low^).

Having demonstrated that the combination of PDT and immunostimulatory NPs reduced the tumor burden of colon cancers in vivo, in a CD8^+^ T cell-dependent manner, we further investigated the anti-tumor immune response elicited by this treatment, by analyzing diverse immune cell populations present in the tumor microenvironment and secondary lymphoid organs. To this end, we inoculated mice with two MC38 or two CT26 tumors, one on each flank, and then treated only one tumor with the combination, as described above (Figure 4A). The day following the second NP administration, the mice were sacrificed, and several organs were collected and prepared for analysis by flow cytometry. In the MC38 model, all treatments induced the infiltration of neutrophils in the treated tumor (Figure 6A), as previously described for PDT [61]. Interestingly, NPs also induced the infiltration of neutrophils in the untreated tumor (Supplementary Materials Figure S6A). The levels of mature (CD86^+^) and inflammatory (Ly6C^high^) myeloid cells have recently been shown to increase in treatment-responsive tumors but not in relapsed tumors that display resistance to treatment [62]. In line with this, we observed an increase in the levels of mature inflammatory myeloid cells and a decrease in non-inflammatory (Ly6C^−^) cells in the treated tumor after treatment with the combination, compared to all other treatments (Figure 6A). In the untreated tumor, the levels of mature inflammatory monocytes were slightly decreased, while the non-inflammatory myeloid cells were increased (Supplementary Materials Figure S6A). These data indicate the ability of the combination to increase mature inflammatory myeloid cells in the treated tumor (but not in the untreated tumor), which, in turn, is reflected by the responsiveness to treatment. In the dLN of MC38 tumor-bearing mice, the NP and combination treatments led to increased populations of CD11b^+^ and DC (Supplementary Materials Figure S6A). In the spleen, the CD11b^+^ population was also increased, whereas the DC population was decreased after the combination treatment (Supplementary Materials Figure S6A). In both the dLN and the spleen, the number of CD4^+^ T cells were decreased, whereas the CD8^+^ T cells were increased (Supplementary Materials Figure S6A). These results indicate that CD11b^+^ cells, including antigen-presenting cells, are increased in the dLN of treated mice, while the CD4^+^/CD8^+^ T cell ratio is skewed to favor CD8^+^ T cells in the dLN and spleen, which corroborate the tumor-specific, CD8^+^ T cell responses that we previously found to be essential for efficacy. We observed a similar trend in the CT26 tumor-bearing mice, in which the combination treatment increased the levels of neutrophils in the treated tumor (Figure 6B) and, to a lesser extent, in the untreated tumor (Supplementary Materials Figure S6B). Furthermore, the combination increased the number of mature inflammatory myeloid cells and decreased the non-inflammatory cells, in both the treated and the untreated tumors. In the dLN and spleens of the CT26 tumor-bearing mice, the combination induced a strong increase in the number of CD11b^+^ cells and DCs (Supplementary Materials Figure S6B). In the dLN, the CD4^+^/CD8^+^ T cell ratio was similar among all treatments (Supplementary Materials Figure S6B); however, in the spleen, the NP and combination treatments shifted this ratio to CD8^+^ T cells, albeit marginally (Supplementary Materials Figure S6B). Together, these data indicate that, in tumor-bearing mice, the combination of PDT and immunostimulatory NPs induces inflammation in the tumor microenvironment, coinciding with greater neutrophil infiltration, as well as higher levels of CD11b^+^ cells and DCs in secondary lymphoid organs. Moreover, this combination appears to skew the CD4^+^/CD8^+^ T cell ratio in favor of CD8^+^ T cells, in line with our previous observation that the efficacy of this combination is dependent on tumor-specific CD8^+^ T cells.

## 4. Discussion

The tumor debulking effects of photodynamic therapy are often insufficient to induce complete and lasting therapeutic efficacy. However, recent studies that exploit the ability of PDT to initiate immune responses, in combination with immunotherapy, show great promise [44,45,46]. In the present study, we combined PDT with immunostimulatory NPs loaded with poly(I:C), R848, and MIP3α, of which the antitumor efficacy of each individual component was previously investigated [20]. We synthesized these biodegradable PLGA NPs, loaded them with immunostimulatory agents, and characterized them, finding favorable physicochemical properties, a lack of inherent cytotoxicity, and retention of biological activities of the encapsulated immunostimulatory agents. Consistent with literature reports on the immunostimulatory activities of PDT on myeloid cells [4,14,63,64,65,66,67], in our studies, PDT-induced tumor cell death led to the upregulation of dendritic cell maturation markers in vitro.

Expanding on our in vitro findings, we explored our therapeutic combination in mice bearing a single tumor. All treatments fully eradicated the MC38 tumors, extended the survival of MC38-tumor-bearing mice, and were highly effective in delaying the growth of CT26 tumors, with the combination being significantly more effective against CT26 than either treatment alone. TC-1 tumors were less responsive to all treatments, with the combination strongly inhibiting tumor growth, compared to either treatment alone, in addition to the control, but not inducing significant gains in survival. Our therapeutic combination performs well, compared to similar strategies that use PDT and immunostimulatory agents. It shows an efficacy equal to or better than PDT combined with photo-thermal therapy (PTT) and TLR9-agonist CpG [66], PDT combined with CpG and a hypoxia inducible factor (HIF) inhibitor [65], CD276-targeted PDT combined with immune checkpoint inhibitors [68], and PDT with magnetic hyperthermia and immune checkpoint inhibitors [69]; all show a tumor growth inhibition at early timepoints after treatment but do not show long term survival (up to 50 days). The differences in the response to PDT-treatment in vivo among the tumor models are further reflected by our observation of upregulation of DC maturation markers in the PDT-treated tumor cells in vitro, whereby MC38 cells exhibited the greatest upregulation of maturation markers, followed by CT26, while TC-1 cells showed only a slight upregulation. These results suggest a link between the propensity of dying PDT-treated tumor cells to upregulate DC maturation markers and the anti-tumor efficacy of PDT.

In the in vivo experiments exploring bilateral tumors, the PDT-nanoparticle combination most effectively reduced the total tumor burden, compared to either treatment alone. The efficacy of our combination is comparable to a study combining PDT with PTT [70], as well as a study combining PDT with PTT and an indoleamine 2,3-dioxygenase inhibitor [67]. However, studies that combine PDT with immune checkpoint inhibitors often show improved results, mostly on the untreated (distal) tumors [44,45,46]. Importantly, in both the unilateral and the bilateral tumor models, the pre-treatment depletion of CD8^+^ cells abrogated the efficacy of the combination treatment. This observation is in line with previously published results [17] and confirms the importance of CD8^+^ cells, in regards to the benefits of this treatment. Furthermore, the ability of the treatment to induce tumor-specific immune responses was investigated by stimulating the splenocytes of treated, tumor-bearing mice ex vivo with the MC38 neoepitopes Adpgk and Rpl18. This revealed an expansion of these tumor-specific CD8^+^ T cells, producing the cytokines IFN-γ and TNF-α in the treated mice, compared to control (untreated) mice, in which the NPs played the largest role (Figure 5). Additionally, the combination induced significantly higher levels of tumor-specific CD8^+^ T cells to TC-1 tumors, compared to either treatment alone. These observations corroborate literature that describes PDT as a modality that facilitates the exposure of previously inaccessible tumor epitopes, to induce and/or enhance tumor-specific immune responses. This ability of PDT to function as an in situ vaccination modality has often been hypothesized; however, it has, to our knowledge, only been shown for exogenous antigens (ovalbumin) [71] and not for cancer neoepitopes (Rpl18 and Adpgk). Although high levels of circulating, tumor-specific T cells to TC-1 (HPV16 E7) have been shown after combining PDT with a specific vaccination using synthetic long peptides for TC-1 [58], we report strongly elevated blood levels of such T cells after the combination with nonspecific immunostimulatory NPs, thereby providing proof to the in situ vaccination ability of PDT when it is combined with a strong adjuvant.

Finally, we evaluated the immunological composition of the tumor microenvironment after the treatment of tumor-bearing mice. Our data show that the combination treatment alters the immunosuppressive tumor microenvironment into a more proinflammatory one, by increasing the presence of mature inflammatory myeloid cells and decreasing the non-inflammatory monocytes in the treated tumor. This observation is in line with other studies combining PDT and immunotherapy, which also show an increased inflammatory state in the tumor after treatment [46,65,66,67,69,72]. Immune checkpoint inhibitors have been shown to enhance the infiltration of CD8^+^ T cells in the untreated (distal) tumor, whereas PDT alone did not [44,45]. Our observations, that local treatment with immune stimulating nanoparticles combined with PDT induces a potent tumor-specific CD8^+^ T cell response, provide a rationale for further enhancing abscopal effects, via the systemic treatment with immune checkpoint inhibitors. The clinical efficacy of immune checkpoint inhibitors is often hampered by the immunosuppressive state present in the tumor microenvironment [73], which we may repolarize to become more proinflammatory after our local nanoparticle treatment. In practice, intravenous injection may also result in the accumulation of the immunostimulatory NP in untreated (distal) tumors. Therefore, a protocol that combines PDT with intravenously administered PLGA-PEG(poly(I:C), R848, MIP-3α) and immune checkpoint inhibitors could be of clinical advantage. Our future studies will explore the potential of such protocols in the treatment of primary and metastatic tumors.

Together, our results show that the combination of PDT and immunostimulatory NPs functions as an in situ vaccination strategy that induces strong, CD8^+^ T cell-dependent, anti-tumor immune responses and elicits abscopal effects. As the benefits of combining classical ablation and immune therapies treatments are increasingly appreciated [74,75,76], the potential of our PDT-nanoparticle combination, that we have presented in this study, may contribute to more effective treatment protocols for solid tumors.

## Figures and Tables

**Table 1:** Physicochemical characterization of the NPs.

Sample	Diameter	ζ Potential (mV)	PDI	Encapsulation Efficiency (% *w*/*w*)			
				NIR	poly(I:C)	R848	MIP3α
NP(NIR + pIC + R848 + MIP3α)-PEG	249.6 ± 85.4	−21.4 ± 4.75	0.178 ± 0.042	62.4 ± 6.9	43.6 ± 8.6	54.2 ± 8.9	59.3 ± 7.3

Physicochemical characterization of the PLGA-PEG NPs containing immunostimulatory agents. The PLGA NPs were characterized as reported previously [20], by dynamic light scattering and zeta potential measurements. The size and zeta potential data represent the mean value ± SD of 10 readings of one representative batch. The concentration of the NIR dye was measured by fluorescence. The concentration of poly(I:C), R848, and MIP3α was determined by RP-HPLC analysis.

## Data Availability

Not applicable.

## Supplementary Materials

The following are available online at https://www.mdpi.com/article/10.3390/pharmaceutics13091470/s1, Figure S1: Synthesis and characterization of PLGA-PEG(poly(I:C), R848, MIP-3α). (A). Size distribution and (B). distribution of the ζ -potential of the PLGA-PEG-NPs used in this study. (C). Maturation of D1DCs after 24 h of incubation with the NP added to correspond to concentrations of 0–5 µg/mL poly(I:C) (light blue bars) compared to 5 µg/mL pure poly(I:C) (black bars), shown as expression of CD40 (left bars) and CD86 (right bars). (D). IL-12P40 expression by D1DCs after 24 h of incubation with the NP at indicated concentration (light blue bars) compared to 5 µg/mL pure poly(I:C) (black bars). (E–G). Toxicity of the NPs to MC38, CT26 and TC-1 after 72 h of incubation at indicated concentrations compared to free poly(I:C) and empty NPs incubated at equal concentrations, determined using the MTS assay, Figure S2: Animal weight of mice bearing unilateral tumors. The weight of animals bearing a single MC38 (A), CT26 (B) or TC-1 (C) tumor after treatment, corresponding to the data in Figure 3, Figure S3: Blood levels of CD8^+^ cells after treatment with CD8-depleting antibodies. Levels of CD8^+^ cells in blood of mice bearing a single MC38 (A) or CT26 (B) tumor in control (untreated) mice and mice that received CD8-depleting antibodies (aCD8), measured 1 day after administering CD8-depleting antibodies, Figure S4: Survival curves of mice bearing bilateral MC38 or CT26 tumors. Immunocompetent mice were inoculated with tumor cells in the right and left flanks and injected with CD8-depleting antibodies one day before tumors became established. When the tumors were established (~125 mm^3^), PDT was performed on one tumor by administration of 20 mg/kg Radachlorin in the tail vein and irradiating with 662 nm light at a drug-to light interval of 6 h and 116 mW/cm^2^ for 116 J/cm^2^. The next morning, animals were injected with NPs at an interval of 2 days for a total of 4 administrations. Survival curves of mice bearing two (A). MC38 tumors (C57BL/6J mice) and (B). CT26 tumors (BALB/c mice), one on each opposite flank, Figure S5: Animal weight of mice bearing bilateral tumors. The weight of animals bearing a two MC38 (A), CT26 (B) tumors on opposite flanks after treatment, corresponding to the data in Figure 4, Figure S6: Analysis of the tumor microenvironment after treatment. Immunocompetent mice were inoculated with cancer cells in the right and left flanks (n ≥ 5). When the tumors were established (~125 mm^3^), PDT was performed on one tumor by administration of 20 mg/kg Radachlorin in the tail vein and irradiating with 662 nm light at a drug-to light interval of 6 h and 116 mW/cm^2^ for 116 J/cm^2^. The next morning, animals were injected with NPs at an interval of 2 days for a total of 2 administrations. The day following the second NP administration, the mice were sacrificed after which the dLN and spleen were collected, processed, and stained for analysis by flow cytometry. Populations are shown in percentages for A). MC38 and B). CT26 tumor-bearing mice. Gating was performed in FlowJo and included only living (7AAD-) CD45.2+ cells. Populations were further gated to include neutrophils (CD11b+Ly6G+), mature inflammatory myeloid cells (CD11b+CD86+Ly6Chi), non-inflammatory myeloid cells (CD11b+Ly6Clow), CD11b (total CD11b+), dendritic cells (DCs, CD11b+CD11chi), CD4 T cells (CD3+CD4+) and CD8 T cells (CD3+CD8+).
