# Supplementary material for: Combining Photodynamic Therapy with Immunostimulatory Nanoparticles Elicits Effective Anti-Tumor Immune Responses in Preclinical Murine Models
Source: Pharmaceutics. 2021 Sep 14;13(9):1470. doi: 10.3390/pharmaceutics13091470 (PMC8465537; doi:10.3390/pharmaceutics13091470)
Supplement: Supplementary file 1 [file pharmaceutics-13-01470-s001.zip › pharmaceutics-1331795-supplementary.pdf]

pharmaceutics

MDPI

Supplementary Materials: Combining Photodynamic Therapy with Immunostimulatory Nanoparticles Elicits Effective Anti-Tumor Immune Responses in Preclinical Murine Models

Ruben V. Huis in 't Veld, Candido G. da Silva, Martine J. Jager, Luis J. Cruz and Ferry Ossendorp

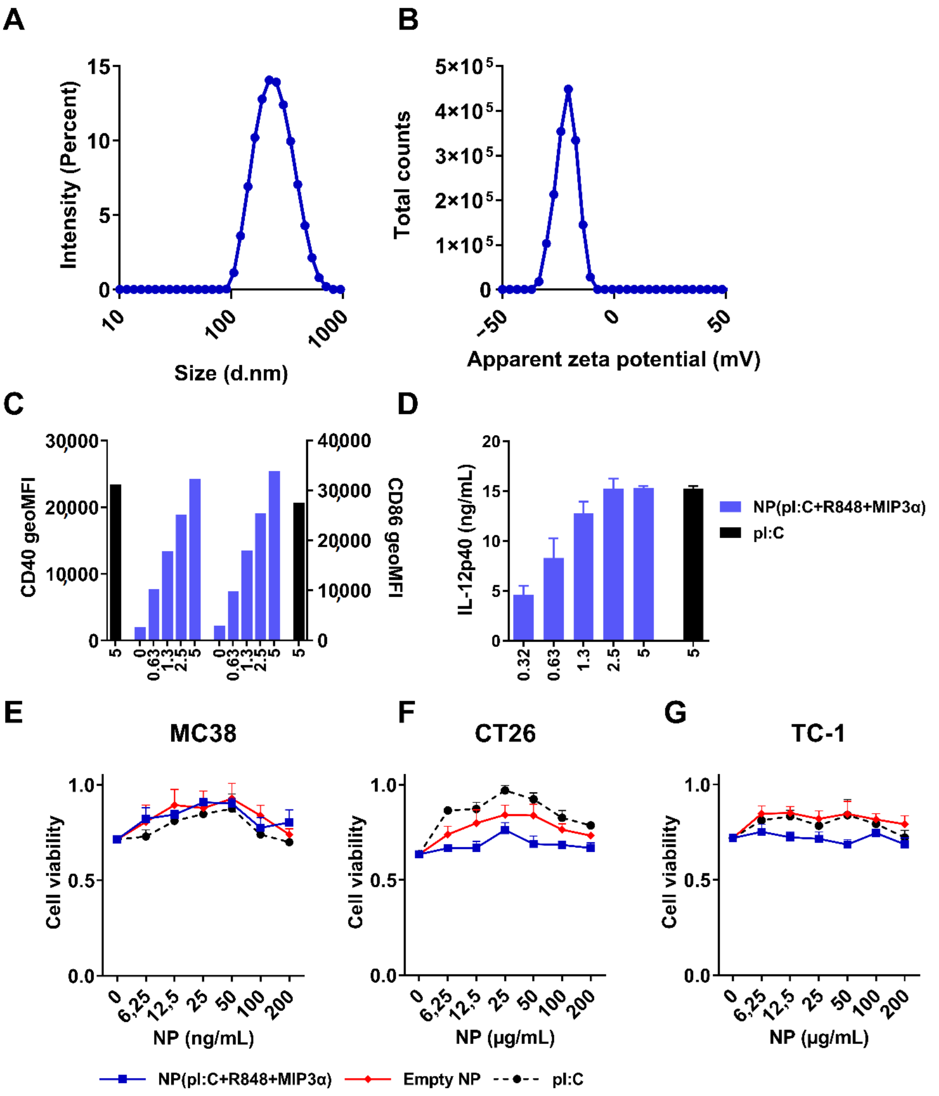

Figure S1. Synthesis and characterization of PLGA-PEG(poly(I:C), R848, MIP-3α). (**A**) Size distribution and (**B**) distribution of the ζ -potential of the PLGA-PEG-NPs used in this study. (**C**) Maturation of D1DCs after 24 h of incubation with the NP added to correspond to concentrations of 0–5 µg/mL poly(I:C) (light blue bars) compared to 5 µg/mL pure poly(I:C) (black bars), shown as expression of CD40 (left bars) and CD86 (right bars). (**D**) IL-12P40 expression by D1DCs after 24 h of incubation with the NP at indicated concentration (light blue bars) compared to 5 µg/mL pure poly(I:C) (black bars). (**E–G**)Toxicity of the NPs to MC38, CT26 and TC-1 after 72 h of incubation at indicated concentrations compared to free poly(I:C) and empty NPs incubated at equal concentrations, determined using the MTS assay. All data shown consist of an average of three independent experiments.

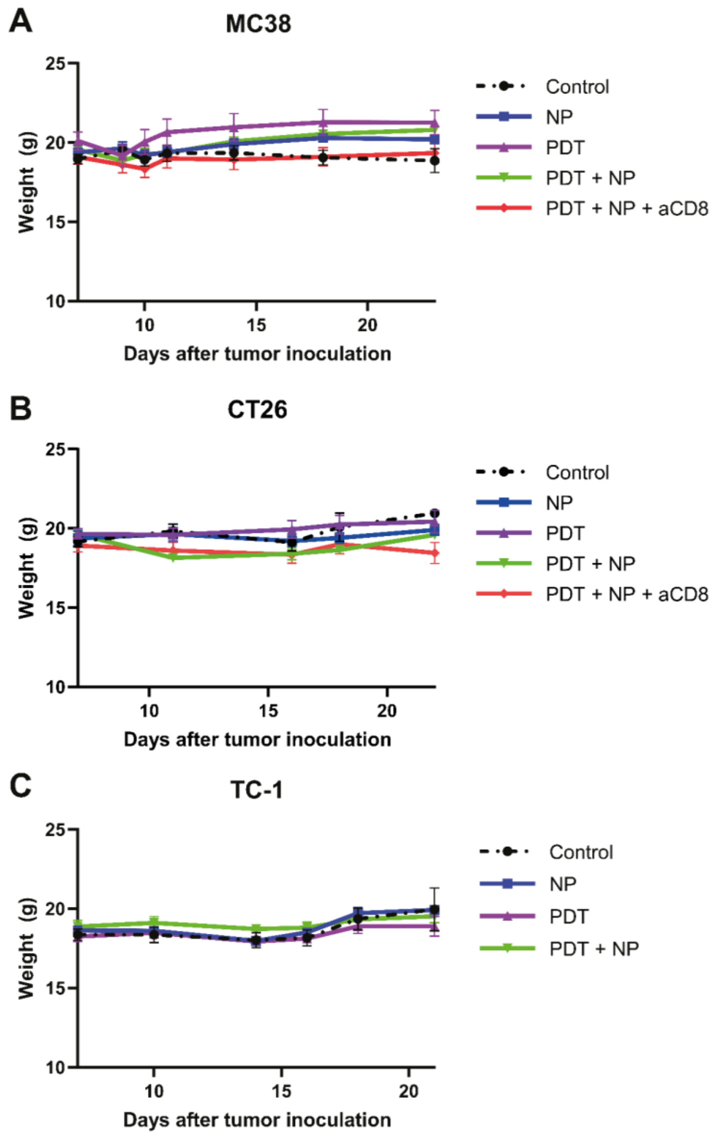

Figure S2. Animal weight of mice bearing unilateral tumors. The weight of animals bearing a single MC38 (**A**), CT26 (**B**) or TC-1 (**C**) tumor after treatment, corresponding to the data in Figure 3.

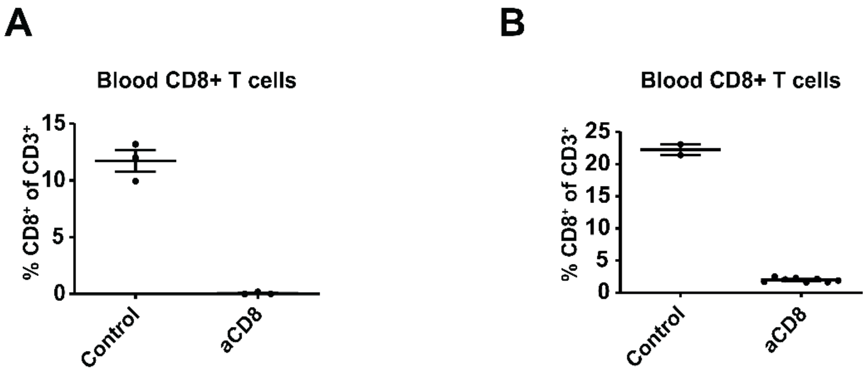

Figure S3. Blood levels of CD8$^+$ cells after treatment with CD8-depleting antibodies. Levels of CD8$^+$ cells in blood of mice bearing a single MC38 (**A**) or CT26 (**B**) tumor in control (untreated) mice and mice that received CD8-depleting antibodies (aCD8), measured 1 day after administering CD8-depleting antibodies.

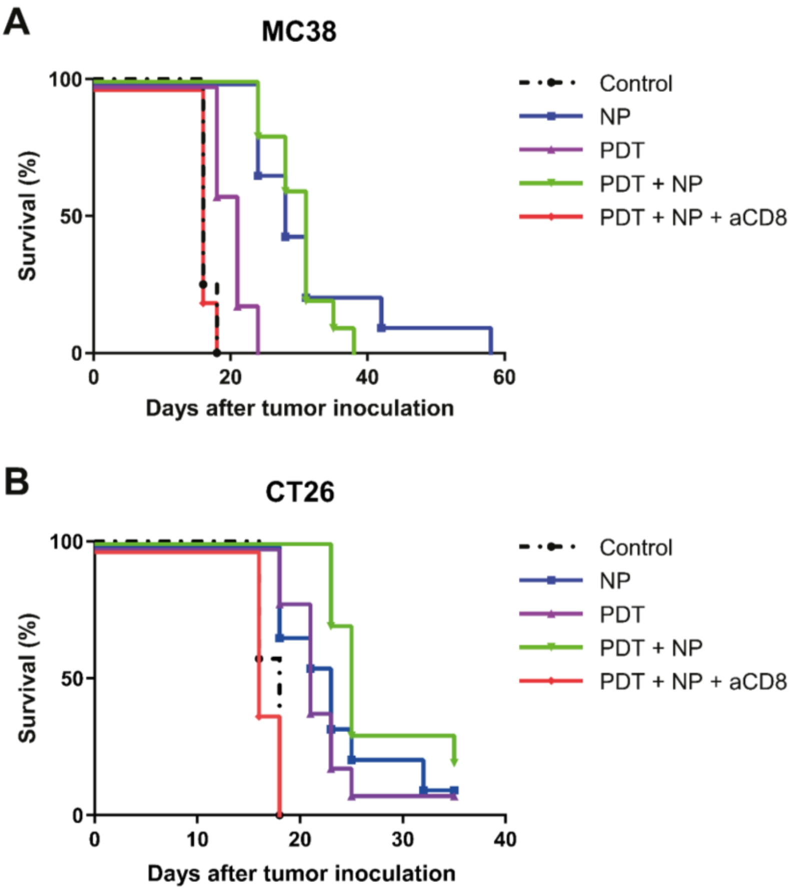

Figure S4. Survival curves of mice bearing bilateral MC38 or CT26 tumors. Immunocompetent mice were inoculated with tumor cells in the right and left flanks and injected with CD8-depleting antibodies one day before tumors became established. When the tumors were established (~125mm^3), PDT was performed on one tumor by administration of 20 mg/kg Radachlorin in the tail vein and irradiating with 662 nm light at a drug-to light interval of 6h and 116 mW/cm^2 for 116 J/cm^2. The next morning, animals were injected with NPs at an interval of 2 days for a total of 4 administrations. Survival curves of mice bearing two (**A**) MC38 tumors (C57BL/6J mice) and (**B**) CT26 tumors (BALB/c mice), one on each opposite flank.

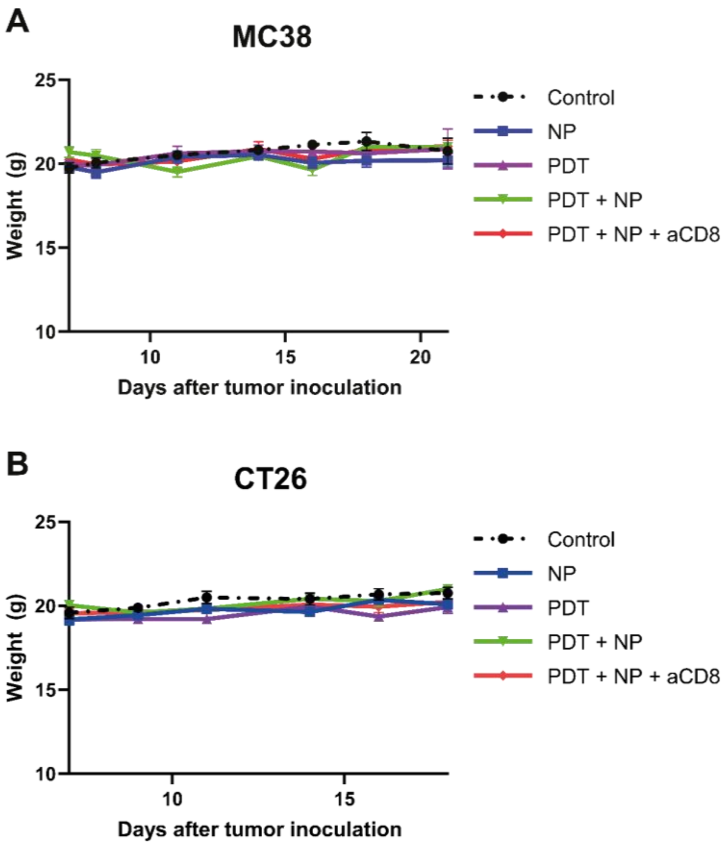

Figure S5. Animal weight of mice bearing bilateral tumors. The weight of animals bearing a two MC38 (**A**), CT26 (**B**) tumors on opposite flanks after treatment, corresponding to the data in Figure 4.

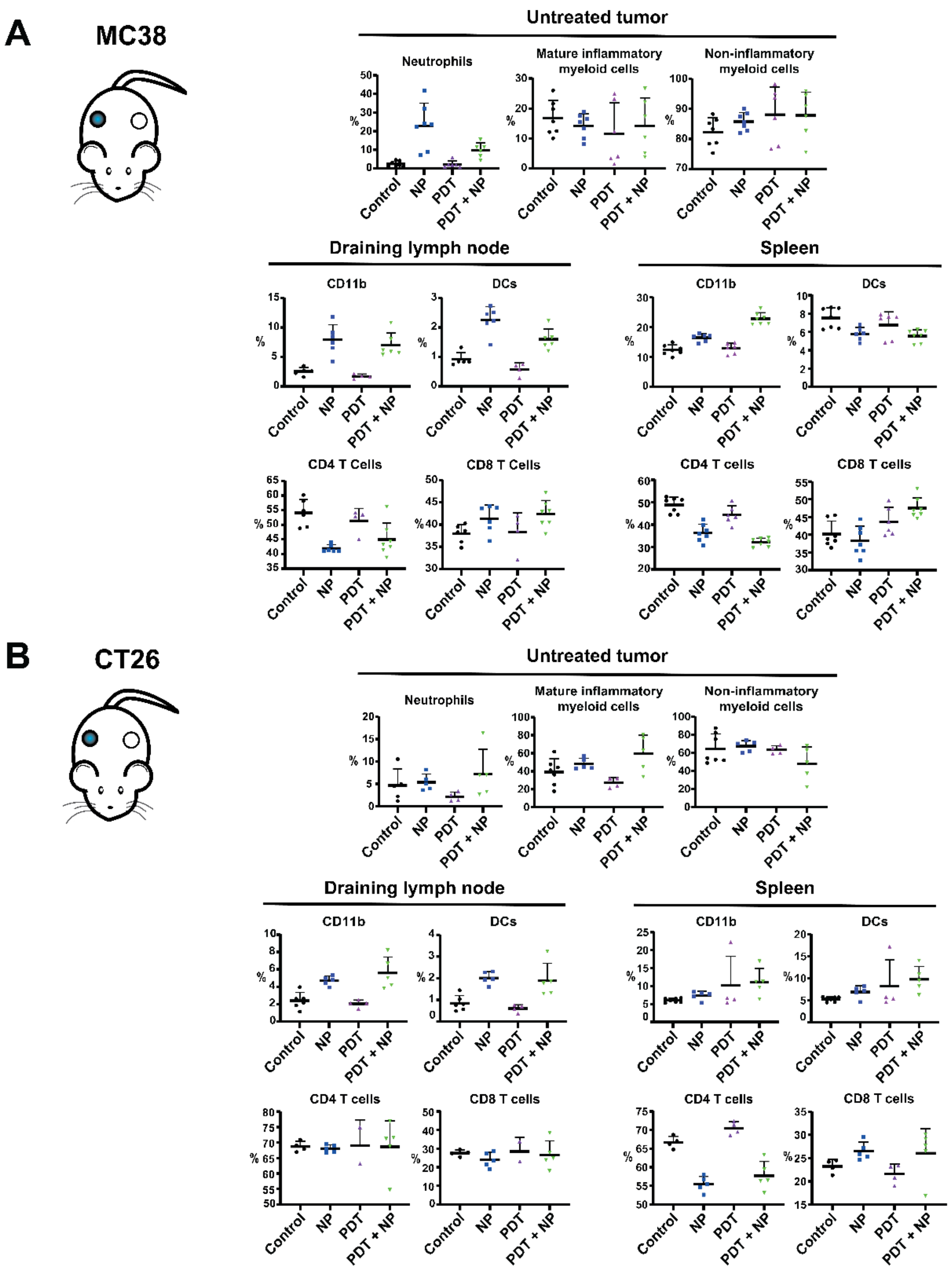

Figure S6. Analysis of the tumor microenvironment after treatment. Immunocompetent mice were inoculated with cancer cells in the right and left flanks ($n \geq 5$). When the tumors were established (~125mm^3), PDT was performed on one tumor by administration of 20 mg/kg Radachlorin in the tail vein and irradiating with 662 nm light at a drug-to light interval of 6h and 116 mW/cm^2 for 116 J/cm^2. The next morning, animals were injected with NPs at an interval of 2 days for a total of 2 administrations. The day following the second NP administration, the mice were sacrificed after which the dLN and spleen were collected, processed, and stained for analysis by flow cytometry. Populations are shown in percentages for (**A**) MC38 and (**B**) CT26 tumor-bearing mice. Gating was performed in FlowJo and included only living (7AAD$^-$) CD45.2$^+$ cells. Populations were further gated to include neutrophils (CD11b$^+$Ly6G$^+$), mature inflammatory myeloid cells (CD11b$^+$CD86$^+$Ly6C^{hi}), non-inflammatory myeloid cells (CD11b$^+$Ly6C^{low}), CD11b (total CD11b$^+$), dendritic cells (DCs, CD11b$^+$CD11c^{hi}), CD4 T cells (CD3$^+$CD4$^+$) and CD8 T cells (CD3$^+$CD8$^+$).
